# Intrapartum human immunodeficiency virus transmission rate in a central hospital in the Western Cape province after universal antiretroviral therapy roll-out

**DOI:** 10.4102/sajid.v35i1.192

**Published:** 2020-12-03

**Authors:** Tian A. van der Merwe, Gert U. van Zyl, Carl J. Lombard, Gerhard B. Theron

**Affiliations:** 1 Department of Obstetrics and Gynaecology, Faculty of Medicine and Health Sciences, Stellenbosch University, Cape Town, South Africa; 2 Division of Medical Virology, Faculty of Medicine and Health Sciences, Stellenbosch University, Cape Town, South Africa; 3 National Health Laboratory Service, Tygerberg Virology, South Africa; 4 Division of Epidemiology and Biostatistics, Department of Global Health, Stellenbosch University, Cape Town, South Africa

**Keywords:** human immunodeficiency virus, HIV, mothers, birth, prevention of mother-to-child transmission, PMTCT, intrapartum

## Abstract

The national human immunodeficiency virus (HIV) mother-to-child transmission rate at 6–10 weeks post-partum was 0.9% in 2016. There is a paucity of data about the intrapartum transmission rate after lifelong antiretroviral therapy was implemented in 2015. We assessed all pregnant women living with HIV who delivered at Tygerberg Hospital in 2017. Positive polymerase chain reactions (PCRs) at birth indicated an *in utero* transmission rate of 0.8%. One infant with a negative PCR at birth tested positive at 6–10 weeks. The intrapartum transmission rate was low (0.08%). About 25% of infants were lost to follow-up after birth.

## Introduction

In South Africa, human immunodeficiency virus (HIV) remains a significant contributor to deaths in children under the age of 5 years, in addition to being the third-leading cause of maternal mortality in the country.^1^ Vertical transmission of HIV from mother to child can occur antenatally, intrapartum or during breastfeeding. The World Health Organization (WHO) is aiming for the elimination of HIV transmission, which increasingly appears to be an achievable goal.^2^

Without any intervention, the perinatal mother-to-child transmission (PMTCT) rate is 25% – 30%.^3^ A randomised controlled trial from the era before the implementation of universal combination antiretroviral therapy (ART) randomised more than 1500 formula-fed infants younger than 48 hours, born to women diagnosed with HIV during labour, into three groups; each group was prescribed a different post-exposure prophylactic ART regimen, namely, zidovudine (AZT) monotherapy, dual AZT and nevirapine (NVP) or a multidrug regimen consisting of AZT, lamivudine and nelfinavir.^4^ The overall mother-to-child transmission (MTCT) rate was 8.5% at 6 weeks post-partum, with an intrapartum transmission rate of 5.7%. The intrapartum transmission rate was greatly reduced in the group who received the multidrug regimen. Factors that were associated with an increased chance of transmission were treatment with AZT mono therapy, an increased HIV viral load (VL) and the concomitant maternal use of illegal substances. In another pre-ART-era trial, by the Pediatric AIDS Clinical Trials Group Study, where AZT was given to mothers in labour only and a single-drug prophylaxis was given to infants, the PMTCT rate was 5%. The only independent risk factor in a multivariate analysis that significantly increased MTCT was the maternal HIV-1 plasma RNA VL counts antenatally and at birth showed low maternal CD4 levels, low p25 antibody levels and chorioamnionitis also increased MTCT.^5^

The WHO PMTCT programme implemented in South Africa in 2015 requires all pregnant and breastfeeding women to be initiated on lifelong ART regardless of their CD4 count (Option B+). The PMTCT rate at 6 weeks was reduced from 3.6% in 2011 to 1.5% in 2016.^6^ This transmission rate has further decreased to 0.7% in 2019.^7^ In a national study identifying barriers to the elimination of PMTCT at national, provincial and district levels, the rate of *in utero* transmission (i.e. infants who were HIV infected at birth) was 0.9%. At provincial and district levels these rates increased to 1.3% and 1.9%, respectively.^8^

In some African countries where WHO Option B+ was implemented, with mothers receiving triple-regimen ART and infants ART prophylaxis, the elimination of PMTCT is almost a reality. A study conducted in south-eastern Nigeria found that none of 182 infants were HIV infected at 6 weeks.^9^ In a similar study carried out in Burkina Faso in 2014, 160 infants of mothers who received triple-regimen ART all tested negative at their 6-week follow-up.^10^ Very low PMTCT will require compliance with ART to maintain an undetectable VL during breastfeeding.^11^

There is a paucity of data about the intrapartum transmission rate in South Africa since universal combination ART (Option B+) has been introduced to pregnant women living with HIV (PWLHIV). A literature search found no data about the present intrapartum transmission rates. Moyo et al. found an intra-uterine transmission rate of 1.1% in 2015 and 2016.^7^ The authors concluded that with an effective PMTCT programme the intra-uterine infections would outnumber the intrapartum infections by three to one. No intrapartum transmission rates were determined. In this study, we aimed to determine the intrapartum transmission rate in a hospital with a high prevalence of HIV following Option B+ roll-out.

*In utero* MTCT is determined by a positive HIV-1 qualitative positive polymerase chain reaction (PCR) at birth. A negative PCR at birth and a positive PCR at 6–10 weeks is suggestive of intrapartum MTCT. Maternal ART may suppress viral replication in infected intra-uterine infants, causing undetectable or indeterminate HIV-1 PCR results at birth.^7^ Late antenatal transmission or early breastfeeding-associated transmission also needs to be considered. A positive PCR result after a negative result at the 6–10 weeks interval indicates MTCT through breastfeeding.^12^ Most of the cumulative transmission through breastfeeding occur in the first 6 months. The Western Cape consolidated guidelines for HIV treatment stipulate that all pregnant women must be tested for HIV at booking, 20 weeks, 32 weeks and again in labour.^13^ Breastfeeding is universally encouraged. The intrapartum HIV transmission rate at Tygerberg Academic Hospital (TBH) in the Western Cape province after the roll-out of universal ART was investigated. The labour ward of TBH provides specialist and sub-specialist levels of care.

## Methods

In this descriptive study, we extracted data from all PWLHIV who delivered their infants at TBH between 01 January and 31 December 2017. The Fetal Evaluation Clinic keeps a register of all PWLHIV who delivered at TBH and includes their infants’ birth HIV-1 PCR. Infants with a positive PCR at birth were recorded to determine the *in utero* MTCT rate. Infants that died *in utero* were also documented. Prescription of dual prophylaxis, consisting of AZT and NVP, for infants at high risk of MTCT was also recorded.

We used the National Health Laboratory Service (NHLS) InterSystems TrakCare^®^ lab viewer, a laboratory information system, to track the infants’ HIV-1 PCR results, taken at 6–10 weeks post-delivery at their various clinics. Results from all clinics and hospitals are nationally accessible on this system, allowing even patients who have relocated to be tracked. Infants with negative PCRs at birth and positive PCRs at 6–10 weeks were used to determine the intrapartum MTCT rate. Either a folder number or a name and date of birth were used to track results. If no birth PCR or 6-week follow-up could be found, infants were regarded as lost to follow-up (LTFU). Variables that may influence MTCT (Table 1) were compared between the LTFU group and the follow-up group to exclude any systematic errors in the interpretation of the results.

**TABLE 1:** Comparison of maternal and neonatal variables between the infants with a follow-up polymerase chain reaction at 6–10 weeks and the infants that were lost to follow-up.

Variable	Follow-up		Lost to follow-up		*p*
	*n*	%	*n*	%	
**Total**	1179	75	392	25	-
**Not on ART**	19	1.6	7	1.8	0.81
**Viral load**	-	-	-	-	0.19
Lower than detectable	770	65.3	242	61.7	-
< 1000 copies/mL	256	21.7	85	21.7	-
≥ 1000 copies/mL	98	8.3	44	11.2	-
**Infant ART prophylaxis**	-	-	-	-	0.64
NVP only	868	73.6	284	72.4	-
NVP and AZT	311	26.4	108	27.6	-
**Delivery route**	-	-	-	-	0.3
Vaginal delivery	504	42.7	156	39.8	-
Caesarean section	675	57.3	236	60.2	-
**CD4 count**	-	-	-	-	0.7
< 200 cells/*μ*L	153	13.0	57	14.5	-
≥ 200 cells/*μ*L	939	79.6	305	77.8	-
No CD4 result	87	7.4	30	7.7	-
**Birth weight of infant**	-	-	-	-	0.21
< 1500 g	99	8.4	36	9.2	-
1500 g – 2499 g	324	27.5	90	23	-
≥ 2500 g	752	63.8	264	67.3	-
**Gestation at delivery**	-	-	-	-	0.01
< 34 weeks	184	15.6	63	16.1	-
34–36 weeks 6 days	220	18.7	48	12.2	-
≥ 37 weeks	771	65.4	281	71.7	-

ART, antiretroviral therapy; NVP, nevirapine; AZT, zidovudine.

Human immunodeficiency virus-1 qualitative PCR testing was performed using the COBAS AmpliPrep/COBAS TaqMan (CAP/CTM) HIV-1 qualitative test (Roche Molecular Systems, Inc., Branchburg, NJ, USA), which detects both HIV-1 DNA and RNA in whole blood or dried blood spots. HIV-1 plasma RNA VL quantification was determined using the CAP/CTM HIV-1 version 2 quantitative test (Roche Molecular Systems, Inc.).

Chi-square tests were used to compare the maternal and neonatal variables in the infants that had a follow-up HIV-1 PCR and those that were LTFU (Table 1).

### Ethical consideration

Ethical clearance to conduct the study was obtained from the Health Research Ethics Committee of the Faculty of Medicine and Health Sciences at Stellenbosch University (clearance number: S 19/01/011).

## Results

**FIGURE 1:**
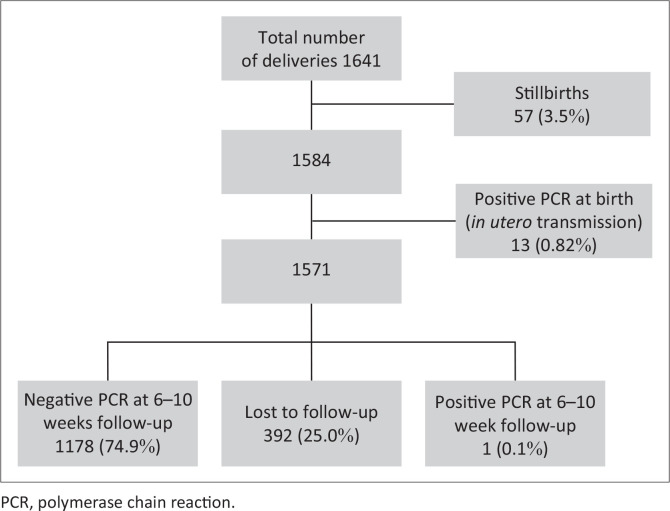
*In utero* and intrapartum transmission rates of infants born to pregnant women living with human immunodeficiency virus at Tygerberg Hospital in 2017.

The total number of babies delivered at TBH in 2017 was 7986.^14^ Of these, 1641 were of PWLHIV (20.5%, Figure 1). Of all deliveries, 520 (6.5%) were stillbirths (SB). SB delivered by PWLHIV amounted to 57 (3.5%). The *in utero* MTCT rate was 0.82%, with 13 of the live-born infants being HIV-positive at birth. No birth PCR could be found in the register or on the NHLS InterSystems Trackcare^®^ for 19 (1.2%) infants, and 373 (23.7%) infants did not have a follow-up PCR done after their birth PCR. The total of LTFU infants was 392 (25.0%). Of the remaining 1178 infants on whom follow-up PCRs were performed, 1177 were negative, and only one baby with an initial negative PCR had a positive PCR at the 6–10 week follow-up, which indicated an intrapartum MTCT rate of 0.08%.

A comparison of the women in the LTFU and follow-up groups (Table 1) revealed them to be remarkably similar. Dual prophylaxis was given to 311 (26.4%) infants in the LTFU group and 108 (27.6%) in the follow-up group. The gestational age at delivery was significantly more advanced in the LTFU group compared to the follow-up group (*p* = 0.01). There were 281 (71.7%) term deliveries (37 weeks or more) in the LTFU group compared to 771 (65.4%) in the follow-up group. No significant differences were found between the groups comparing the very low birth weight infants (< 1500 g) and low birth weight infants (< 2500 g).

The mother of the infant who was most likely infected intrapartum was a 25-year-old primigravida who attended an antenatal clinic for the first time at 26 weeks of gestation. She was diagnosed with HIV the previous year and was initiated on ART. Her VL was undetectable at her first antenatal visit, which was 4 months before delivery, and the CD4 count was 110 cells/*μ*L. Her VL was not repeated at delivery, but a VL performed 2 months after delivery was 8874 copies/mL. She went into spontaneous labour after presented with antepartum bleeding and an episode of severe hypertension at 36 weeks. She was allowed to continue with spontaneous labour because the conditions of both the mother and the baby were satisfactory. Her membranes ruptured spontaneously 24 hours before delivery. The second stage of labour lasted 30 minutes with an uncomplicated delivery and intact perineum; the birth weight of the baby was 2080 g and the Apgar scores at 1, 5 and 10 minutes were 7, 9 and 9, respectively. Meconium stained liquor was noticed and the infant required continuous positive airway pressure resuscitation, but was transferred with the mother to the ward. The infant received post-exposure prophylaxis with NVP for 6 weeks. The mother’s choice of feeding was breastfeeding. The infant’s birth HIV-1 PCR was negative; however, at the 10-week follow-up the PCR was positive. HIV infection was confirmed with a subsequent positive HIV-1 PCR at 12 weeks and 5 days of age. The plasma HIV-1 RNA load was 85 707 copies/mL at 7 months of age and the mother’s VL at the same time was undetectable.

## Discussion

Data from the pre-ART era indicated that intrapartum MTCT rate was 5.7%.^4^ There is, however, minimal data available for the intrapartum MTCT rate after the roll-out of WHO Option B+. This study found the reassuring result of a low intrapartum transmission rate of 0.08%, with only one baby in the cohort possibly infected during labour and delivery.

The only case where intrapartum transmission most likely occurred was a premature delivery, with a coinciding prolonged rupture of the membranes, which are both risk factors of *in utero* and intrapartum transmission. It is unclear from the notes whether the mother was using her ART at the time and her VL at delivery was not determined. The fact that her VL 2 months after delivery was high possibly indicates that her VL at delivery was also high, which would increase the risk of intrapartum MTCT. Because of her presumed undetectable VL, the baby was only prescribed 6 weeks of NVP. Late antenatal transmission or the rare possibility of transmission through breast milk also needs to be considered.

A limitation of this study is the LTFU rate of 25%. The only significant difference between the infants in the LTFU and follow-up groups was that the LTFU group had a significantly more advanced gestational age at delivery. No significant differences between the other variables known to be associated with PMTCT were found. The 25% LTFU rate is concerning and an important finding of this study. Health education about the importance of attending the Baby Clinic for the follow-up PCR should be emphasised. It also indicates the need for qualitative research to understand why PLWHIV avoid follow-up PCR testing of their infants.

The results of this study indicate the need for a large sample size to be included in future research on intrapartum transmission. Case investigations where intrapartum transmission occurred will be most helpful in determining the risk factors of transmission. The low intrapartum MTCT rate in this study is reassuring and indicates that South Africa is on track towards eliminating PMTCT. Consistent quality assurance regarding the PMTCT programme and data collection is mandatory. The NHLS InterSystems TrakCare^®^ could be used to trace infants LTFU and to track PCR results, regardless of where the 6–10 week follow-up visit was done. The early initiation of ART in infants has been shown to reduce morbidity and mortality and to improve neurodevelopmental outcomes; therefore, it is of paramount importance to educate mothers on the significance of follow-up visits.^15^

## Data Availability

Data sharing is not applicable to this article as no new data were created or analysed in this study.
